# Live imaging-based assay for visualising species-specific interactions in gamete adhesion molecules

**DOI:** 10.1038/s41598-022-13547-w

**Published:** 2022-06-10

**Authors:** Kohdai P. Nakajima, Clari Valansi, Daisuke Kurihara, Narie Sasaki, Benjamin Podbilewicz, Tetsuya Higashiyama

**Affiliations:** 1 Division of Biological Science, Graduate School of Science, Nagoya University, Furo-cho, Chikusa-ku, Nagoya, Aichi 464-8602 Japan; 2 Department of Biology, Technion-Israel Institute of Technology, 32000 Haifa, Israel; 3 JST, PRESTO, Nagoya, Japan; 4 Institute of Transformative Bio-Molecules (WPI-ITbM), Nagoya University, Furo-cho, Chikusa-ku, Nagoya, Aichi 464-8601 Japan; 5 Institute for Human Life Innovation, Ochanomizu University, 2-1-1 Ohtsuka, Bukyo-ku, Tokyo, 112-8610 Japan; 6 Department of Biological Sciences, Graduate School of Science, The University of Tokyo, 7-3-1 Hongo, Bunkyo-ku, Tokyo, 113-0033 Japan

**Keywords:** Cell biology, Molecular biology

## Abstract

Successful gamete fusion requires species-specific membrane adhesion. However, the interaction of adhesion molecules in gametes is difficult to study in real time through low-throughput microscopic observation. Therefore, we developed a live imaging-based adhesion molecule (LIAM) assay to study gamete adhesion molecule interactions in cultured cells. First, we modified a fusion assay previously established for fusogens introduced into cultured cells, and confirmed that our live imaging technique could visualise cell–cell fusion in the modified fusion assay. Next, instead of fusogen, we introduced adhesion molecules including a mammalian gamete adhesion molecule pair, IZUMO1 and JUNO, and detected their temporal accumulation at the contact interfaces of adjacent cells. Accumulated IZUMO1 or JUNO was partly translocated to the opposite cells as discrete spots; the mutation in amino acids required for their interaction impaired accumulation and translocation. By using the LIAM assay, we investigated the species specificity of IZUMO1 and JUNO of mouse, human, hamster, and pig in all combinations. IZUMO1 and JUNO accumulation and translocation were observed in conspecific, and some interspecific, combinations, suggesting potentially interchangeable combinations of IZUMO1 and JUNO from different species.

## Introduction

Sexually reproducing organisms commonly reproduce via the union of different sex cells. Fertilisation is the final process of sexual reproduction; it is achieved through the recognition, adhesion, and fusion of gametes in processes regulated by fertilisation molecules on gametes. Fertilisation molecules have been identified in several species. For example, in mammals, sperm that have passed through the zona pellucida approach the egg and ultimately fertilise it through interactions of fertilisation molecules, such as IZUMO1 on the sperm and CD9 and JUNO on the egg^1–3^. Mutants of IZUMO1 and JUNO are completely infertile and CD9 mutant drastically reduced fertility. Co-crystal structure analysis has demonstrated that IZUMO1 and JUNO interact as adhesion molecules, and that species-specific binding depends on the conserved amino acid sequence, e.g., W148A of IZUMO1 and W62A of JUNO, on the interaction surface^4,5^, although the machinery required for membrane fusion during fertilisation remains unclear. To date, only IZUMO1 and JUNO have been identified as a gamete adhesion molecule pair. A recent study also reported that the sperm proteins SPACA4/6, TMEM95, SOF1, FIMP and DCST1/2 are required for mammalian fertilisation^6–11^.

In flowering plants, two immotile sperm cells are conveyed by the pollen tube to the female gametes, and the adhesion molecule GAMETE EXPRESSED 2 (GEX2) and fusogen GENERATIVE CELL-SPECIFIC 1/HAPLESS 2 (GCS1/HAP2) on the sperm cell membrane promote fertilisation with the two female gametes, i.e. the egg cell and central cell^12–14^, in a unique fertilisation process referred to as double fertilisation. GEX2 has a filamin-like domain similar to the Ig-like domain contained in IZUMO1. The green alga *Chlamydomonas reinhardtii* has no GEX2 orthologue; however, FUS1, which contains a filamin-like domain, is expressed on plus-type cells^15^. Recently, FUS1 partner molecule, Minus Adhesion Receptor 1 (MAR1), was identified, which was required not only for gamete adhesion but for focused localization of GCS1/HAP2 at the mating structure^16^. GCS1/HAP2 does not exist in mammals, and fusogens have not been identified in mammalian gametes. Current studies are seeking to identify the complete set of fertilisation molecules in several species. Missing partner molecules and the function and action mechanisms of known fertilisation molecules require investigation to understand the precise molecular mechanisms of fertilisation.

Species-specific and -preferential interactions among fertilisation molecules also require further investigation. Some proteins involved in mammal male–female interactions have been suggested to be involved in species-specific interactions. Ovulated eggs are surrounded by the zona pellucida; the zona pellucida glycoprotein ZP2 is required for species-specific sperm–egg binding through the zona pellucida in mice and humans^17^. Yanagimachi et al. found that golden hamster zona-free oocytes could be fertilised with sperm of various species (hamster test)^18^. The glycosylphosphatidylinositol (GPI)-anchored protein Bouncer is required for species-specific sperm entry into the egg, and enables cross-species fertilisation between medaka and zebrafish through gene swapping^19^. Extracellular protein interactions were exploited in the AVidity-based Extracellular Interaction Screening (AVEXIS) assay, demonstrating conspecific interaction between IZUMO1 and JUNO in mouse cells; the results suggested that hamster JUNO can interact with human, mouse, and pig IZUMO1^20^, consistent with the crossing result obtained by Yanagimachi et al.^21^. However, in flowering plants, gamete interaction appears to be less critical for species-specific fertilisation than the progamic phase of pollen-pistil interactions^22–24^.

Genetic loss-of-function experiments with phenotypic analysis have revealed that fertilisation-related molecules are required for gamete adhesion or fusion. However, the precise molecular interactions driving cell membrane adhesion and fusion remain to be elucidated. Recent gain-of-function experiments have investigated the molecular functions of membrane proteins. For example, nematode specific proteins EFF-1 and AFF-1 have been shown to fuse nematode, insect, mammalian and viral membranes^25–29^. More recently, the muscle-specific protein Myomaker was transfected with Myomerger (also called Myomixer and Minion) into fibroblasts to investigate their biochemical function in cell-membrane fusion^30^. Gain-of-function experiments in heterologous cell systems are powerful tools for the elucidation of molecular functions with characteristic activities. Combining this technique with live-cell imaging would allow us to visualise molecular dynamics and functions in real time.

Various assays were developed previously to investigate molecular adhesiveness of adhesion molecule pairs. Cell adhesion assay and cell aggregation assay are for qualification and quantification of adhesion molecules by evaluating whether transfected suspension cells form cell mass^31,32^. Cell-oocyte assay and sperm-binding assay are for analysing genes involved in gamete interactions by using sperms or oocytes and cultured cells^33,34^. Chalbi et al. tested the adhesion between oocytes and cultured cells by dual-pipette assay^35^. As mentioned above, Bianchi et al. identified JUNO by AVEXIS assay^3^, which were also used to test the interaction between TMEM95 and JUNO^9^. These assays could evaluate interaction of adhesion molecules, but the interaction of the gametic adhesion molecule pair IZUMO and JUNO was not directly and simultaneously visualised in living cells. A novel live-imaging based assay that can detect interaction of adhesion molecule pairs would contribute to elucidate the molecular dynamics and function of molecules involved in gamete adhesion. Here we developed a system to analyse the interaction of gametic adhesion molecules in cultured animal cells by live-cell imaging. For this gain-of-function approach, we modified a fusion assay developed by Valansi et al.^36^. The fusion assay has never previously been used to evaluate gamete adhesion molecule function. We confirmed that cell–cell fusion of BHK cells expressing fusogens could be captured by our live-cell imaging technique using a disc scanning confocal system embedded within the incubator. Then, we introduced adhesion molecules to explore their effects in cultured cells, which lead us to develop a live imaging-based adhesion molecule (LIAM) assay. LIAM assay was shown to be useful for the screen of species-specificity in gamete adhesion molecules IZUMO1 and JUNO.

## Results

### Fusogen promotes cell–cell fusion in our live-cell imaging system

We aimed to develop a system to study fertilisation molecules in animal cultured cells by live imaging. Various cultured animal cell lines, such as COS-7 and HEK293T, have been used for transfection and expression in previous fertilisation molecule studies; for example, molecular adhesiveness between IZUMO1 and JUNO was shown by mixing gametes and transfected cultured-cells^3,33,34^. In this study, because we focused on cell migration and morphology for frequent contact of cells under microscopy, we selected a line of motile BHK cells that did not spontaneously adhere or fuse with each other (Fig. S1). BHK cells were also used in the original fusion assay^36^. We investigated whether cell fusion activity could be detected with known fertilisation molecules using our live-cell imaging technique by transfecting BHK cells with RFPcyto as a negative control, and *GCS1/HAP2* to evaluate fusion rates. Ectopic expression of fusogens sometimes indicates toxicity to cells or the whole organism^26,27,36,37^; therefore, tight regulation was required to ensure expression at the preferred times. For this purpose, we used a mifepristone-inducible system as used in the fusion assay^36^.

**Figure 1:**
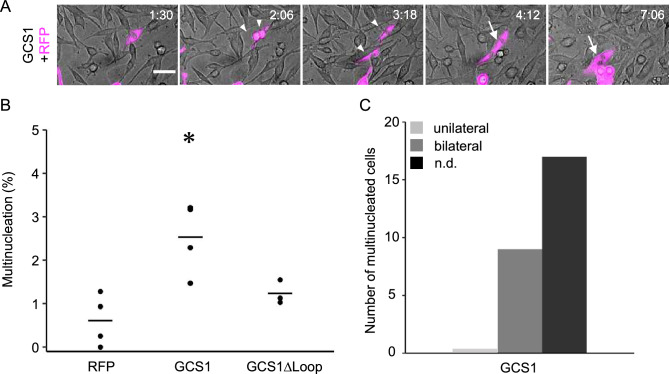
Quantification of multinucleation using live imaging. (**A**) Time-lapse images from a fusion assay. Baby hamster kidney (BHK) cells were transfected by plasmids for expression of red fluorescent protein (RFP) (magenta) and GCS1 (co-transfection). Arrowheads and arrows indicate contacting and fused cells, respectively. Time (h:min) after the start of observation is shown (see Video 1). Bars: 20 µm. (**B**) Multinucleation frequency of BHK cells determined by the expression of GCS1 and GCS1∆Loop. The use of mutant proteins GCS1∆Loop is explained in detail in the text. Dots indicate multinucleated cells among cultured cells, determined using a fusion assay; bars indicate the average values. Dunnett’s test was used to compare each gene to the negative control. *P < 0.05. (**C**) Dependency of cell–cell fusion on co-transfected RFP expression. Bars represent the numbers of multinucleated cells, determined by fusion of RFP-labelled and non-labelled cells (unilateral fusion, light grey bars), fusion of RFP-labelled cells (bilateral fusion, dark grey bars), and fusion of non-labelled cells (not determined, black bars; all cells in this category began to express RFP after fusion).

To capture cell–cell fusion by live imaging, we used a Nipkow disc scanning confocal system embedded within the incubator to monitor cells in 4.0 mm × 4.0 mm areas (tiling of each 810 µm × 810 µm area) every 6 min under stable low-phototoxicity conditions (Fig. 1). In each captured video, 130–476 cells expressing RFPcyto were monitored. When the gamete fusogen GCS1/HAP2 (*Arabidopsis thaliana* GCS1/HAP2; AtGCS1/HAP2) was expressed, cell–cell fusion was observed (Fig. 1A, arrows; Video 1). At 12 h after induction, the fusion rate increased significantly, to 2.2 ± 0.4% (n = 4; *P* < 0.05; Dunnett’s test; Fig. 1B), compared to the fusion rate of the negative control in which only RFPcyto was expressed (0.6 ± 0.5%; n = 4; Fig. 1B). To confirm that cell–cell fusion depended on the fusogen activity of the expressed proteins, we transfected the loop-deletion *GCS1/HAP2* (*GCS1/HAP2∆loop*). Amino acids 166–178 in AtGCS1 represent a hydrophobic loop structure, which is necessary for membrane fusion^38^. The fusion rate was 1.2 ± 0.2% (n = 3), which was not significantly different from the negative control (Fig. 1B). This result suggested that fusogen-dependent cell–cell fusion was visualised by live imaging.

Our live imaging system is useful to monitor expression of a fluorescent marker upon cell–cell fusion. The dependency of individual fusion on the expression of transfected genes from 4 to 12 h after expression induction is shown in Fig. 1C. In experiments for wild-type GCS1/HAP2, a combination of two cells labelled with red fluorescent protein (RFP) was more likely to fuse than a combination of labelled and non-labelled cells. This is partly due to the frequent fusion of daughter cells after cell division. Our result is consistent with the previous report that GCS1/HAP2, as well as EFF-1, bilaterally promoted cell–cell fusion in the fusion assay^27,36^. Together, these results suggest that our live imaging technique is compatible with a modified fusion assay for the study of fusogen functions in cultured cells.

### Adhesion molecules accumulate at the contact interfaces of adjacent cells

**Figure 2:**
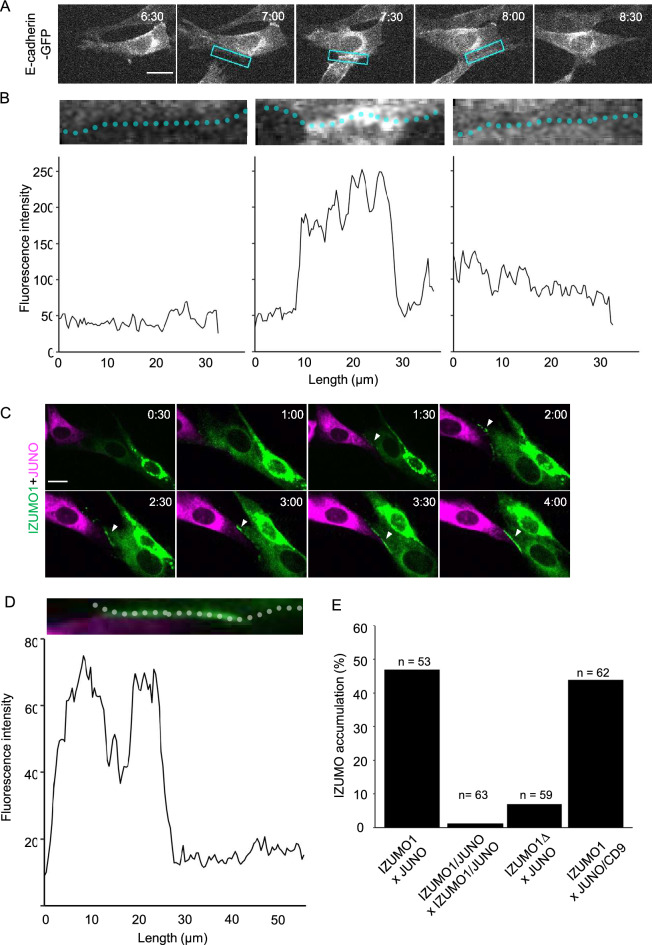
Adhesion molecules accumulated at the contact interface of adjacent cells. (**A**) Time-lapse images of E-cadherin-expressing cells. Box indicates the region where cell membrane fluorescent intensity was measured in (**B**). Time (h:min) since the start of observation is shown (see Video 2). Bar: 20 µm. (**B**) Fluorescence intensity profile of cells shown at the top. (Left panel) Cells prior to contact; (middle) cells immediately after contact; (right) cells immediately after detachment. (**C**) Time-lapse images of IZUMO1-expressing cells (green) and JUNO expressing-cells (magenta). Time (h:min) since the start of observation is shown (see Video 3). Bar: 20 µm. Arrowheads indicate IZUMO1 accumulation at the contact site. (**D**) Fluorescence intensity profile of IZUMO1 in a cell shown at the top. (**E**) IZUMO1 accumulation rate for various treatment combinations. Numbers indicate contacted cell pairs observed by live imaging.

First, we transfected the somatic adhesion molecule E-cadherin into BHK cells. Cadherins have been shown to play a role in Ca^2+^-dependent cell–cell adhesion^39^. The transfection of a single cadherin gene can potentially induce interaction of cadherin expressed in different cells (homophilic adhesion), eliminating the necessity of introducing an adhesion molecule pair such as IZUMO1 and JUNO (heterophilic adhesion). Cadherin was visualised by translational fusion with GFP, and no toxicity was observed despite the lack of an inducible gene expression system for cadherin in this experiment. E-cadherin-expressing BHK cells in our system did not adhere to neighbouring cells to show aggregation. E-cadherin appeared to be localised at the cell membrane and secretory pathways of the cell, showing no polarisation (Fig. 2A). However, upon temporary contact between E-cadherin-expressing cells, E-cadherin tended to accumulate at the contact interface (Fig. 2A, 7:30; Video 2); fluorescent intensity of E-cadherin-GFP increased specifically at the interface of cell contact (Fig. 2B, 7:30). This accumulation disappeared as cells detached (Fig. 2B, 8:00).

Next, we introduced mouse IZUMO1 and JUNO to determine whether a similar temporal accumulation at the contact interface of adjacent cells might occur even in heterophilic adhesion molecules. We developed a mixing assay, in which cells expressing IZUMO1 and JUNO were mixed at 4 h after independent transfection and were incubated for 20 h. IZUMO1 and JUNO were designed as translational fusion molecules according to previous reports^33,40^ with fluorescent proteins at the C-terminal and the N-terminal (just after the signal peptide), respectively. At 4–12 h after the induction of IZUMO1 and JUNO expression by mifepristone, we found that IZUMO1 temporally accumulated at the contact interfaces of JUNO-expressing cells (47% of contacting cells, n = 53; Fig. 2C–E; Video 3).

We examined whether IZUMO1 accumulation is dependent on the interaction of IZUMO1 and JUNO expressed in adjacent cells. We at first confirmed that by immunostaining, these proteins are reaching the cell surface. Untransfected BHK cells showed the affinity to neither α-IZUMO1 nor α-JUNO antibodies (Fig. S2). Immunostaining in non-permeabilised and permeabilised BHK cells expressing IZUMO1 or JUNO visualised both proteins localized at the cell surface even before the cell–cell contact (Fig. S3). When IZUMO1 and JUNO were expressed simultaneously in the same cell by co-transfection, neither molecule accumulated on the cell surface (0%, n = 63; Fig. 2E). Consistently, IZUMO1 was not localized at the cell surface in the co-transfected cells (Fig. S4). Next, we constructed a mutant IZUMO1 lacking the β-hairpin region (IZUMO1∆) and examined the interaction between IZUMO1∆ and JUNO. The central β-hairpin region of IZUMO1 is required for JUNO binding, as has been shown by co-crystal structure analysis^4,5^. When IZUMO1∆- and JUNO-expressing cells were mixed, accumulation of IZUMO1 was rarely observed (7% of cases, n = 59; Fig. 2E). We also co-transfected JUNO with CD9, an egg tetraspanin required for gamete fusion^2^, to determine whether CD9 modulates IZUMO1 accumulation. When IZUMO1- and JUNO/CD9-expressing cells were mixed, IZUMO1 accumulation and frequency were unchanged, consistent with the finding that CD9 is independent of IZUMO1-JUNO mediated gamete adhesion^2,41^ (44%, n = 62; Fig. 2E). These results demonstrate the usefulness of this assay for visualising the dependence of adhesion molecule interactions on the affinity of introduced adhesion molecule pairs. We named this assay system the live imaging-based adhesion molecule (LIAM) assay, which we propose for the examination of transfected adhesion molecules at cell contact sites.

### Accumulation and translocation of adhesion molecules in the LIAM assay depended on critical amino acids

**Figure 3:**
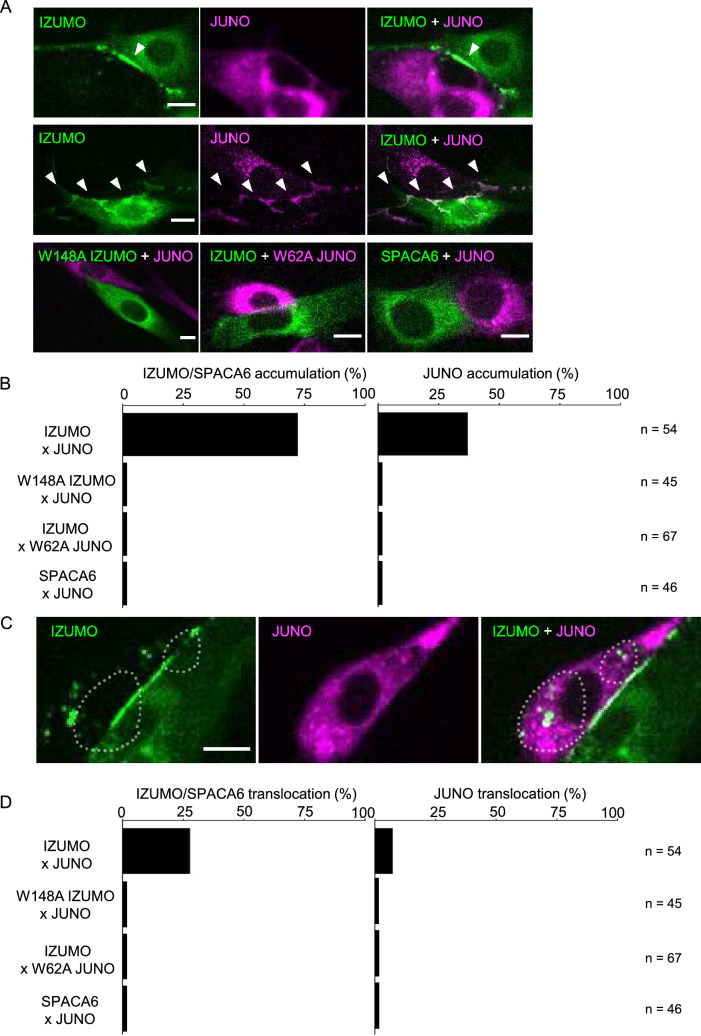
The accumulation and translocation of IZUMO1 and JUNO depended on their amino acid sequences. (**A**) LIAM assay with mouse IZUMO1/SPACA6 and JUNO. Mixing of IZUMO1-expressing cells (green) and JUNO-expressing cells (magenta) induced IZUMO1 accumulation (top panels) or simultaneous accumulation of IZUMO1 and JUNO (middle panels) (see Video 4). Arrowheads indicate accumulation of IZUMO1 or JUNO. Point mutations of amino acids critical for binding, i.e., W148A IZUMO1 (bottom left) and W62A JUNO (bottom middle), impaired accumulation. The sperm membrane protein SPACA6 (bottom right) did not induce accumulation of JUNO. Bars: 10 µm. (**B**) IZUMO1/SPACA6 and JUNO accumulation rates. (**C**) Translocation of IZUMO1 to an adjacent cell. Note that translocated signals (enclosed by dashed lines) continued to act as florescence foci. Bars: 10 µm. (**D**) IZUMO1/SPACA6 and JUNO translocation rates.

Subsequent experiments were conducted using high-throughput analysis in a non-inducible gene expression system (cytomegalovirus promoter), because we found that the inducible gene expression system was unnecessary for cadherin, IZUMO1, and JUNO (translational fusion with fluorescent proteins). Prior to contact between mouse IZUMO1- and JUNO-expressing cells, these adhesion molecules were distributed evenly throughout the cell, as also occurs in the inducible gene expression system. Accumulation was observed upon cell contact; however, both IZUMO1 and JUNO showed accumulation (Fig. 3A; Video 4). IZUMO1 accumulation was observed in 72% of contacting cells (39/54; Fig. 3B), whereas JUNO accumulation was observed in only 37% (20/54; Fig. 3B). IZUMO1 and JUNO accumulation were always simultaneously observed in the same cell pairs (opposite sides of a single contact site) when JUNO accumulation was observed.

To determine whether accumulation was dependent on the amino acid residues of IZUMO1 and JUNO for molecular interaction, we performed a LIAM assay using mutant IZUMO1 and JUNO, according to previous crystal structure analysis results^4,5^ (Fig. 3A, Table S1). When mouse mutant IZUMO1 W148A-expressing cells were mixed with mouse JUNO-expressing cells, accumulation was not observed (0% of 45 contacting cells; Fig. 3B). When mouse IZUMO1-expressing cells and mouse mutant JUNO W62A-expressing cells were mixed, accumulation was again not observed (0% of 67 contacting cells; Fig. 3B). Prior to cell contact, IZUMO1 and JUNO expression and localisation were unchanged by these point mutations; both IZUMO1 W148A t and JUNO W62A proteins were localized at the cell surface (Fig. 3A, Fig. S5). Next, we tested the sperm transmembrane protein SPACA6, which is required for fertilisation^7,8^, instead of IZUMO1, which shows structural similarity with SPACA6^7^ (Fig. S6, Table S1), and found that neither SPACA6 nor JUNO accumulated (Fig. 3A). These results indicate that IZUMO1 and JUNO accumulation depends on amino acids critical for their interaction, according to the LIAM assay.

In 38% (15/39) of the cells showing IZUMO1 accumulation, IZUMO1 was found to translocate to the plasma membrane of the opposite cell (Fig. 3C). Several fluorescent foci of IZUMO1 or JUNO on the opposite cell were observed upon the detachment of contacting cells (Video 5). IZUMO1 was translocated to JUNO-expressing cells in 27% of contacting cells (15/54; Fig. 3D) and JUNO was translocated to IZUMO1-expressing cells in 7.4% of contacting cells (4/54; Fig. 3D). This translocation of IZUMO1 and JUNO was unilateral, not bilateral, in each cell pair, and translocation was selectively observed for either IZUMO1 or JUNO in each cell pair. In addition, IZUMO1 or JUNO translocation was always observed after IZUMO1 accumulation, but not necessarily after JUNO accumulation, perhaps due to the difficulty associated with visualising JUNO accumulation on the cell membrane.

This translocation was dependent on the amino acid sequence of IZUMO1 and JUNO required for the interaction (Fig. 3D). When mutant IZUMO1 W148A or mutant JUNO W62A was used, neither translocation not accumulation was observed (Fig. 3D). SPACA6 and JUNO also did not induce translocation (Fig. 3D). In subsequent experiments, this translocation of adhesion molecules was also monitored in the LIAM assay.

### Species specificity in JUNO and IZUMO1 interactions suggested by the LIAM assay

**Figure 4:**
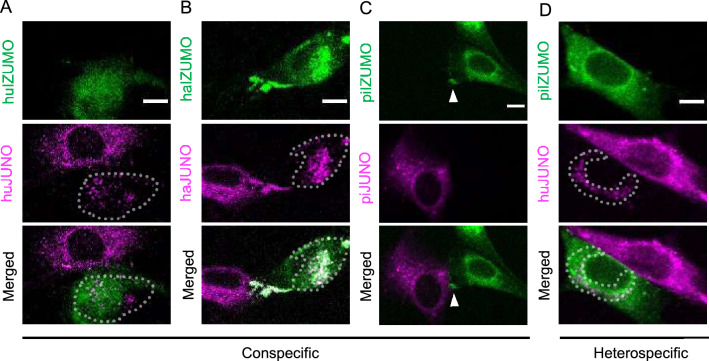
(**A**–**D**) Translocation and accumulation of conspecific and heterospecific combinations. Dashed lines surround molecular translocation sites (see Video 5). Arrowhead in (**C**) indicates IZUMO1 accumulation; no translocation was observed in this conspecific combination (pig). Bars: 10 µm.

Using the LIAM assay, we investigated the species specificity of IZUMO1 and JUNO from mouse, human, hamster, and pig (Fig. 4; Tables 1, 2, 3, 4; Table S2). Among these four mammalian species, mice are most closely related to hamsters, and humans and pigs are phylogenetically close to each other^42^. Molecular phylogenetic analysis results for IZUMO1 and JUNO proteins were consistent with the phylogenetic relationships among these four species (Figs. S7–S9). The mouse IZUMO1 protein shares 61%, 52%, and 50% homology (identity) with hamster, human, and pig IZUMO1 proteins, respectively (Figs. S7, S9). In contrast, the mouse JUNO protein shares 74%, 68%, and 65% homology with hamster, human, and pig JUNO proteins, respectively (Figs. S8, S9). These four species were selected because species specificity has been observed biochemically in their IZUMO1 and JUNO interactions, in some but not all combinations, using the AVEXIS assay^3^. In the AVEXIS assay, IZUMO1 and JUNO interaction was shown in all conspecific combinations, hamster JUNO and all other three species, and mouse JUNO and human IZUMO1 but not in human JUNO and mouse IZUMO1.

**Table 1:** IZUMO1 accumulation in conspecific and heterospecific cell combinations. Numbers in brackets are the numbers of contacted cell-pairs for each combination.

JUNO	IZUMO1			
	Mouse	Human	Hamster	Pig
Mouse	72% (54)	0.0% (50)	33% (36)	0.0% (64)
Human	50% (38)	27% (51)	0.0% (58)	48% (63)
Hamster	78% (40)	0.0% (46)	77% (57)	13% (84)
Pig	0.0% (26)	0.0% (38)	0.0% (37)	24% (41)

**Table 2:** JUNO accumulation in conspecific and heterospecific cell combinations. Numbers in brackets are the numbers of contacted cell-pairs for each combination.

JUNO	IZUMO1			
	Mouse	Human	Hamster	Pig
Mouse	37% (54)	2.0% (50)	25% (36)	0.0% (64)
Human	0.0% (38)	29% (51)	3.4% (58)	49% (63)
Hamster	0.0% (40)	0.0% (46)	16% (57)	7.1% (84)
Pig	0.0% (26)	0.0% (38)	0.0% (37)	0.0% (41)

**Table 3:** IZUMO1 translocation in conspecific and heterospecific cell combinations. Numbers in brackets are the numbers of contacted cell-pairs for each combination.

JUNO	IZUMO1			
	Mouse	Human	Hamster	Pig
Mouse	28% (54)	0.0% (50)	0.0% (36)	0.0% (64)
Human	0.0% (38)	0.0% (51)	0.0% (58)	3.2% (63)
Hamster	0.0% (40)	0.0% (46)	44% (57)	0.0% (84)
Pig	0.0% (26)	0.0% (38)	0.0% (37)	0.0% (41)

**Table 4:** JUNO translocation in conspecific and heterospecific cell combinations. Numbers in brackets are the numbers of contacted cell-pairs for each combination.

JUNO	IZUMO1			
	Mouse	Human	Hamster	Pig
Mouse	7.4% (54)	0.0% (50)	0.0% (36)	0.0% (64)
Human	0.0% (38)	65% (51)	0.0% (58)	32% (63)
Hamster	0.0% (40)	0.0% (46)	28% (57)	0.0% (84)
Pig	0.0% (26)	0.0% (38)	0.0% (37)	0.0% (41)

We examined all combinations of IZUMO1 and JUNO from mouse, human, hamster, and pig cells using the LIAM assay. We began with conspecific combinations. Mouse IZUMO1 and JUNO showed accumulation in 72% (39/54; Fig. 3; Table 1) and 37% (20/54; Fig. 3; Table 2) of contacting cells, respectively. Translocation of mouse IZUMO1 and JUNO was also observed, in 27% (15/54; Table 3; Fig. 3C) and 7.4% (4/54; Table 4) of contacting cells, respectively. This finding is consistent with previous AVEXIS assay results^3^, which showed that mouse IZUMO1 and JUNO show relatively strong binding. In this study, we observed IZUMO1 accumulation as often as JUNO accumulation in human IZUMO1 × human JUNO interactions (IZUMO1 accumulation: 27%, 14/51, Table 1; JUNO accumulation: 29%, 15/51, Table 2). In this combination, IZUMO1 translocation was not observed (Table 3), whereas JUNO translocation was frequently observed (64%, 33/51, Table 4, Fig. 4A). In hamster IZUMO1 × hamster JUNO, IZUMO1 and JUNO accumulation (IZUMO1: 77%, 44/57, Table 1; JUNO: 16%, 9/57, Table 2) and translocation (IZUMO1: 44%, 25/57, Table 3; JUNO: 28%, 16/57, Table 4, Fig. 4B) were observed, consistent with mouse IZUMO1 and JUNO. In pig IZUMO1 × pig JUNO interactions, we observed IZUMO1 accumulation at a low rate (24%, 10/41, Table 1, Fig. 4C). Conspecific interaction of IZUMO1 and JUNO was confirmed for all combinations, and the frequencies of IZUMO1 and JUNO accumulation and translocation differed among species.

Next, we examined heterospecific interaction between mouse IZUMO1 and human, hamster, and pig JUNO. When mouse IZUMO1-expressing cells and human JUNO-expressing cells were mixed, mouse IZUMO1 accumulated at the cell interface (50%, 19/38; Table 1), but neither JUNO accumulation nor IZUMO1/JUNO translocation was observed (Tables 2, 3, 4). When mouse IZUMO1-expressing cells and hamster JUNO-expressing cells were mixed, only mouse IZUMO1 accumulated (78%, 31/40, Table 1), consistent with mouse IZUMO1 and human JUNO. When mouse IZUMO1-expressing cells and pig JUNO-expressing cells were mixed, we observed neither accumulation nor transference. These results indicate that the translocation of adhesion molecules to opposite cells correlated well with conspecific interactions.

Next, we examined heterospecific interaction between human IZUMO1 and mouse, hamster, and pig JUNO. Neither accumulation nor translocation was detected in any combination (Tables 1, 2, 3, 4). However, human IZUMO1 has been reported to interact with hamster JUNO^20^. Therefore, we tried another cell HEK293T, a derivative of human embryonic kidney 293 cell line, although cell-motility was lower compared to that of BHK cells. When mouse IZUMO1-expressing HEK293T cells and mouse JUNO-expressing HEK293T cells were mixed as a positive control, accumulation of both IZUMO1 and JUNO was detected at the interface of the cells (IZUMO1: 89%, 66/74; JUNO: 55%, 41/74; Fig. S10A, Table S3). Next, human IZUMO1-expressing HEK293T cells and hamster JUNO-expressing HEK293T cells were mixed. Accumulation of both human IZUMO1 and hamster JUNO was detected (IZUMO1: 9.3%, 10/107; JUNO: 2.8%, 3/107; Fig. S10B, Table S4).

When hamster IZUMO1-expressing cells and mouse JUNO-expressing cells were mixed, IZUMO1 and JUNO accumulation were detected, although at lower rates than for conspecific combinations (mouse IZUMO1 × mouse JUNO, hamster IZUMO1 × hamster JUNO) (Tables 1 and 2). This result is consistent with the crossing results reported by Yanagimachi et al.^18^ and those of a previous AVEXIS assay^3^. No translocation was observed (Tables 3 and 4). Finally, we combined pig IZUMO1-expressing cells with mouse, human, and hamster JUNO-expressing cells. When pig IZUMO1-expressing cells and mouse JUNO-expressing cells were mixed, neither accumulation nor translocation was observed (Tables 1, 2, 3, 4). However, when pig IZUMO1-expressing cells and human JUNO-expressing cells were mixed, IZUMO1 and JUNO accumulation was detected. Unexpectedly, translocation of both IZUMO1 and JUNO was detected in this heterospecific combination of pig IZUMO1 and human JUNO (Tables 1, 2, 3, 4, Fig. 4D). This strongly positive result was obtained using our LIAM assay, but has not been tested previously using other assays.

## Discussion

In this study, we developed the LIAM assay to study gamete adhesion molecules in cultured animal cells. BHK cell line was the best among cell lines we tested for its motility but another cell line HEK293T was also shown to be available. The LIAM assay represents a powerful complement to the fusion assay^36^ for microscopic examination of the functions of fertilisation molecules in various organisms under highly controllable conditions. As previously observed for fusogens including plant GCS1/HAP2 and nematode EFF-1, the functions of the mammalian adhesion molecule pair IZUMO1 and JUNO were assessed at the contact interfaces of adjacent cells. Two phenotypes of adhesion molecule dynamics, i.e., accumulation and translocation, were detected by the LIAM assay (Fig. 3). The accumulation of homophilic and heterophilic somatic adhesion molecules^39,43,44^, as well as gamete IZUMO1 or JUNO^3,33,35^, has been reported at the interface of adhered cultured cells; however, our live imaging-based assay suggested that temporal contact was sufficient to induce the intracellular accumulation of adhesion molecules in non-aggregating cultured cells. Our results showed that IZUMO-accumulation was more frequently observed than JUNO-accumulation (Tables 1, 2, 3, 4) although both proteins were similarly detected at the cell surface by immunostaining before cell-contact (Fig. S3). Inoue et al.^33^ reported that JUNO accumulates at the very early phase of sperm-egg fusion. Oligomerization of IZUMO, as well as exclusion of JUNO, have also been reported in sperm-egg fusion^33^. The kinetics and dynamics of IZUMO-JUNO interaction remain to be elucidated, and LIAM assay would contribute to it by visualising their interaction in real time. The translocation in LIAM assay is also an interesting phenomenon that may be caused by protein extraction from the membrane of the adjacent cell during contact. Unidirectional translocation (i.e. selective translocation of IZUMO1 or JUNO in a single cell pair) and stable foci of translocated proteins suggest that some cell activity, such as endocytosis, trogocytosis^45^, or exosome internalization within a cell pair, is involved in the translocation and compartmentalisation of adhesion molecules, depending on the molecule combination. Biological significance of the translocation is still unclear; however, it is interesting that oocyte membrane transfers to sperm after their contact^46^, adding to exosome perception by the sperm before the contact^46,47^. CD9-independet trogocytosis has been suggested for the direct transfer^46^. LIAM assay might contribute to understand molecular and membrane dynamics and its mechanism at the egg-sperm interface.

Accumulation and translocation were strictly dependent on the structures of the tested molecules (Fig. 3). Single amino acid mutations in IZUMO1 or JUNO, which are critical for their interaction, abolished these signals completely. Other loop and domain deletions, as well as the swapping of IZUMO1 and SPACA6, also resulted in drastic decreases in these signals (Figs. 2 and 3). These results suggest that the LIAM assay successfully detected interactions within the adhesion molecule pairs. Consistently, IZUMO1 and JUNO that had simultaneously accumulated within a cell pair were localised together across the contact site (Fig. 3). The frequencies of accumulation and translocation detected by the LIAM assay were strongly correlated with conspecific combinations in which direct interaction of IZUMO1 and JUNO have been shown biochemically using an AVEXIS assay^3^ (Tables 1, 2, 3, 4). This was also consistent with the detection of some heterospecific but compatible combinations, such as mouse sperm IZUMO1 and human egg JUNO^34,35^ or hamster egg JUNO^3^ (Table 1). The application of other analysis techniques, such as fluorescence resonance energy transfer (FRET), would provide insights into the interaction of adhesion molecules observed in our LIAM assay; combining the LIAM assay with FRET would be a very powerful method for visualising molecular interactions in real time, which has not been attempted for gamete interactions in any organisms to date.

The advantages of the LIAM assay include higher throughput to identify potential molecule pairs showing heterophilic and homophilic adhesion activity among many combinations. After confirming conspecific interaction of IZUMO1 and JUNO from mouse, human, hamster, and pig, we identified a candidate combination for strong heterospecific binding: pig IZUMO1 and human JUNO. Among the tested four species, human and pig possess relatively close IZUMO1 and JUNO (Fig. S9). Evaluation of the adhesiveness of pig sperm and a human egg would be of interest, and to our knowledge has not been reported. The LIAM assay showed positive results for hamster JUNO with a wide range of IZUMO1 from mouse, pig, hamster, and human (in HEK293T cells but not in BHK cells; Tables 1, 2, 3, 4 and Fig. S10), which is largely consistent with previous reports that hamster zona-free eggs can be fertilised by sperm of various mammals^21^, and that hamster JUNO can interact with IZUMO1 from various species^20^. It may be possible to determine why hamster JUNO is compatible with IZUMO1 from various mammals in future studies, via detailed amino acid swapping analyses performed using the LIAM assay.

An anticipated direction of in vitro studies using LIAM and fusion assays is reconstitution of the gamete fertilisation machinery in cultured cells, which would be a powerful tool for the study of fertilisation molecules as a machinery complex. In LIAM assay, we did not observe cell–cell fusion, consistent with the previous report that ectopic expression of adhesion molecules is not sufficient for cell–cell fusion of cultured somatic cells^48^. Reconstitution of the fertilisation machinery complex under microscopy is a promising direction for future research to clarify these molecular dynamics and functions. In mammals, partner molecules for the sperm proteins SPACA4/6, TMEM95, SOF1, FIMP, DCST1/2, as well as a fusogen, remain elusive^6–11^. The identification of a complete set of fertilisation molecules, which is in progress in some organisms including *Chlamydomonas*^16^, would contribute significantly to reconstitution in cultured cells or liposomes.

## Methods

### Cell culture and DNA transfection

BHK-21 cells were used in this study (RCB1423; RIKEN Cell Bank, Tsukuba, Japan). BHK cells were grown and maintained in Dulbecco’s modified Eagle’s medium (DMEM; Wako, Osaka, Japan) containing 10% foetal bovine serum (FBS). Cells were cultured at 37 °C in 5% CO_2_. Plasmids were transfected into cells using 20 µL jetPRIME (PolyPlus-transfection, Illkirch-Graffenstaden, France) in 200 µL for every 8-well chambered coverglass (IWAKI, Tokyo, Japan).

### Plasmid construction

*Arabidopsis thaliana GCS1*/*HAP2* coding sequence (CDS) fragments were amplified from pSN30 (a gift from Dr. Shiori Nagahara, Nagoya University) by polymerase chain reaction (PCR) using the primers listed in Supplemental Table 5. *Caenorhabditis elegans* *eff-1*, mouse *IZUMO1*, *Juno*, *Cd9*, or *Spaca6* CDS fragments were amplified from cDNA in each organism. Human, hamster, and pig *IZUMO1* and *Juno* were synthesised artificially (Integrated DNA technologies, Tokyo, Japan). Mouse E-*cadherin* CDS fragments were derived from mouse E-cadherin green fluorescent protein (GFP; 67937; Addgene, Watertown, MA, USA). To visualise IZUMO1, SPACA6, and CD9, the C-terminus of these fragments were fused to fluorescent protein Venus or mTurquoise2 sequences by PCR and then cloned into pGENE B or replaced with an E-cadherin-GFP insert after double digestion with restriction enzymes using the Gibson assembly (NEB, Ipswich, MA, USA). To visualise JUNO, mCherry was inserted just after signal peptide in JUNO^33^. W148A IZUMO-Venus and mCherry-W62A JUNO was made by self-ligation based on IZUMO-Venus and mCherry-JUNO. For inducible expression using mifepristone in BHK cells, we used the GeneSwitch System (Invitrogen, Waltham, MA, USA).

### Microscopy and image acquisition for live cell imaging

Time-lapse images used to detect fusing cells and their interaction were obtained using a spinning disk confocal system (CellVoyager CV1000; Yokogawa Electric, Tokyo, Japan) equipped with 405-, 488-, and 561-nm diode lasers. The incubator equipped with the microscope was set at 37 °C and 5% CO_2_. Confocal images were acquired using 10 × (numerical aperture [NA], 0.40; working distance [WD], 3.1 mm; 10 × UPLSAPO; Olympus, Tokyo, Japan) and 40 × (NA, 0.95; WD, 0.188 mm; 40 × UPLSAPO; Olympus) dry objective lenses. The exposure time was 100 ms for Venus, mCherry, and mTurquoise2. Fluorescence was acquired through band-pass (BP) filters: BP 447/60 for mTurquoise2, BP 525/50 for Venus, and BP 617/73 for mCherry. Image analyses were performed using CV1000 software (Yokogawa Electric) and the Fiji online tool (http://fiji.sc/) was used to adjust the brightness and contrast.

### Fusion analysis

We modified the method by Valansi et al*.*^36^ To evaluate fusion rates, we co-transfected BHK cells with pGENE and pSWITCH. One day prior to transfection, BHK cells were cultured at 5.0 × 10^4^ cells/mL. At 4 h after transfection, expression vectors were induced by addition to DMEM containing 10% FBS and 10^−4^ mM mifepristone. The timing of observation was earlier than the method by Valansi et al*.*^36^ (18 h post-induction) to capture the fusion process: at 3–4 h post-induction, images of the cells were acquired every 6 min for 12 h, using the CV1000 system at a magnification of 10 × to record cell–cell fusion. Approximately 4.0 mm × 4.0 mm square were observed by tiling of each 0.8 mm × 0.8 mm square (25 images in total), wherein 130–476 cells expressing RFPcyto were observed. Firstly, we counted the transfected living cells and evaluated expression efficiency. The expression efficiency was defined as the ratio between expressed living cells (Ec) and living cells (Lc) in 0.4 mm^2^ area (0.2 mm × 0.2 mm square × 10 points observed at a magnification of 40 ×), as follows: % expression efficiency = Ec/Lc × 100. The expression efficiency was calculated as 25 ± 1.6% (n = 3). The fusion rate was defined as the ratio between the number of fused living cells (Fc) and expressed living cells (Ec), as follows: % Fusion = Fc/Ec × 100. Each experiment was repeated at least three times.

### LIAM assay

For interaction experiments, plasmids were transfected into BHK cells. At 4 h post-transfection, the cells were washed with DMEM without FBS and detached using 0.05% trypsin–EDTA. BHK cells were resuspended in DMEM with 10% FBS, and equal amounts of cells were mixed and incubated for 20 h in an 8-well chambered coverglass (IWAKI). After incubation, cell images were acquired every 6 min for 12 h using the CV1000 system at a magnification of 40 × to record interactions. Multi-points, i.e., 10–15 points in each well, were sequentially observed. At each point, 0–7 combinations of cells were observed to contact. The total number of contacting cell-combinations in each observation (sum of the 10–15 points) is shown in Supplementary Tables 1 and 2. Adhesion was defined as accumulation at the interface of the cell membrane. We calculated the accumulation and translocation rates as the proportion of cells showing accumulation or translocation among all contacting cells, as follows: % Accumulation or translocation = cells showing accumulation or translocation/contacting cells × 100. Each experiment was repeated at least three times.

### Statistical analyses

For multinucleation analysis, at least three independent technical replicates were performed. We used Dunnett’s test to compare the means of transfected genes with those of the negative control (RFP). Significant differences were evaluated at *P* < 0.05.

## Supplementary Information

Supplementary Information.

Supplementary Video 1.

Supplementary Video 2.

Supplementary Video 3.

Supplementary Video 4.

Supplementary Video 5.

Supplementary Legends.

## Data Availability

The datasets generated during and/or analysed during the current study are available from the corresponding author on reasonable request.
