# Author Correction: Live imaging-based assay for visualising species-specific interactions in gamete adhesion molecules

**DOI:** 10.1038/s41598-025-08993-1

**Published:** 2025-07-10

**Authors:** Kohdai P. Nakajima, Clari Valansi, Daisuke Kurihara, Narie Sasaki, Benjamin Podbilewicz, Tetsuya Higashiyama

**Affiliations:** 1 Division of Biological Science, Graduate School of Science, Nagoya University, Furo-cho, Chikusa-ku, Nagoya, Aichi 464-8602 Japan; 2 Department of Biology, Technion-Israel Institute of Technology, 32000 Haifa, Israel; 3 JST, PRESTO, Nagoya, Japan; 4 Institute of Transformative Bio-Molecules (WPI-ITbM), Nagoya University, Furo-cho, Chikusa-ku, Nagoya, Aichi 464-8601 Japan; 5 Institute for Human Life Innovation, Ochanomizu University, 2-1-1 Ohtsuka, Bukyo-ku, Tokyo, 112-8610 Japan; 6 Department of Biological Sciences, Graduate School of Science, The University of Tokyo, 7-3-1 Hongo, Bunkyo-ku, Tokyo, 113-0033 Japan

Correction to: *Scientific Reports* 10.1038/s41598-022-13547-w, published online 10 June 2022

The original version of the Article contained an error in Figure 2. As a result of an error during figure assembly, the 6:30 image in Figure 2A was mistakenly a duplication of the 8:30 image.

The original Figure 2 and accompanying legend appear below.

**Fig. 2:**
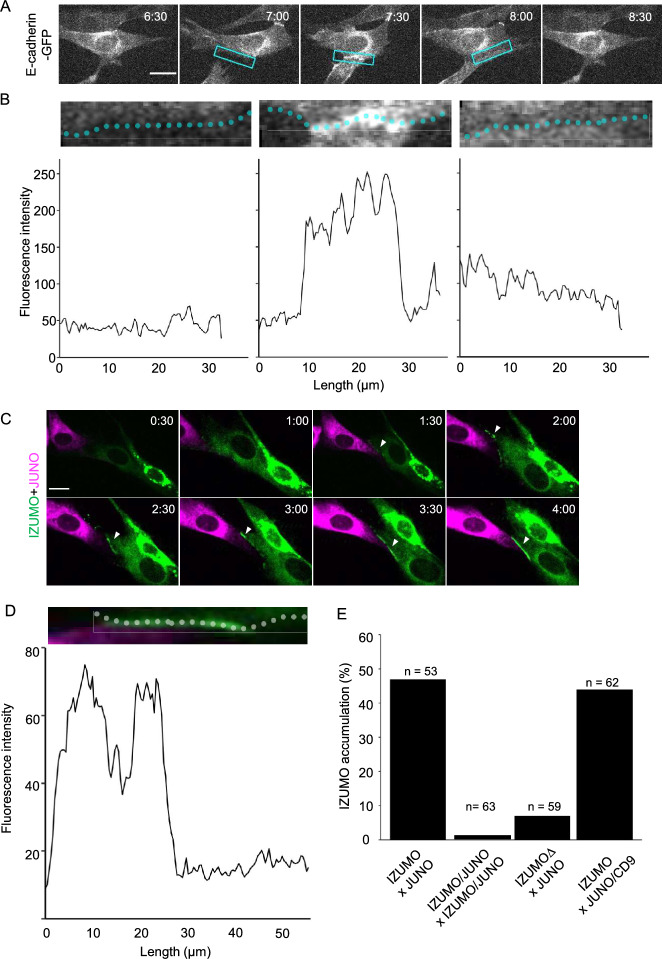
Adhesion molecules accumulated at the contact interface of adjacent cells. (**A**) Time-lapse images of E-cadherin-expressing cells. Box indicates the region where cell membrane fluorescent intensity was measured in (**B**). Time (h:min) since the start of observation is shown (see Video 2). Bar: 20 µm. (**B**) Fluorescence intensity profile of cells shown at the top. (Left panel) Cells prior to contact; (middle) cells immediately after contact; (right) cells immediately after detachment. (**C**) Time-lapse images of IZUMO1-expressing cells (green) and JUNO expressing-cells (magenta). Time (h:min) since the start of observation is shown (see Video 3). Bar: 20 µm. Arrowheads indicate IZUMO1 accumulation at the contact site. (**D**) Fluorescence intensity profile of IZUMO1 in a cell shown at the top. (**E**) IZUMO1 accumulation rate for various treatment combinations. Numbers indicate contacted cell pairs observed by live imaging.

The original Article has been corrected.

