# Supplementary material for: Live imaging-based assay for visualising species-specific interactions in gamete adhesion molecules
Source: Sci Rep. 2022 Jun 10;12:9609. doi: 10.1038/s41598-022-13547-w (PMC9187738; doi:10.1038/s41598-022-13547-w)
Supplement: Supplementary file 1 — Supplementary Information. [file 41598_2022_13547_MOESM1_ESM.pdf]

Supplementary Information

Live imaging-based assay for visualising species-specific interactions in gametic adhesion molecules

Kohdai P. Nakajima[1], Clari Valansi[2], Daisuke Kurihara[3,4], Narie Sasaki[1,5], Benjamin Podbilewicz[2], Tetsuya Higashiyama[1,4,6]

[1]Division of Biological Science, Graduate School of Science, Nagoya University, Furo-cho, Chikusa-ku, Nagoya, Aichi 464-8602, Japan

[2]Department of Biology, Technion- Israel Institute of Technology, Haifa 32000, Israel

[3]JST, PRESTO, Nagoya, Japan

[4]Institute of Transformative Bio-Molecules (WPI-ITbM), Nagoya University, Furo-cho, Chikusa-ku, Nagoya, Aichi 464-8601, Japan

[5]Institute for Human Life Innovation, Ochanomizu University, 2-1-1 Ohtsuka, Bukyo-ku, Tokyo 112-8610, Japan

[6]Department of Biological Sciences, Graduate School of Science, the University of Tokyo, 7-3-1 Hongo, Bunkyo-ku, Tokyo 113-0033, Japan

Supplementary Methods

Cell culture and DNA transfection

COS-7 cells, HeLa cells and HEK293T cells were used in this study. HEK293T cells were grown and maintained in Dulbecco's modified Eagle's medium (DMEM; Wako, Osaka, Japan) containing 10% foetal bovine serum (FBS). Cells were cultured at 37°C in 5% CO_2. Plasmids were transfected into cells using 20 µL jetPRIME (PolyPlus-transfection, Illkirch-Graffenstaden, France) in 200 µL for every 8-well chambered cover glass (IWAKI, Tokyo, Japan).

Molecular phylogenetic analysis

Phylogenetic trees for IZUMO1 and JUNO of mouse, human, hamster, and pig were constructed using the neighbour-joining method in the MEGA X program[42]. Amino acid sequences for entire IZUMO1 proteins were used as follows: mouse, *Mus musculus* (Uniprot accession no. Q9D9J7); human, *Homo sapiens* (Q8IYV9); golden hamster, *Mesocricetus auratus* (A0A1U7QS27); pig, *Sus scrofa* (A0A287BMD8), and opossum, *Monodelphis domestica* (H9H653). Amino acid sequences for entire JUNO proteins were used as follows: mouse, *M. musculus* (Q9EQF4); human, *H. sapiens* (A6ND01); golden hamster, *M. auratus* (A0A1U7RC80); pig, *S. scrofa* (F1STK4), and opossum, *M. domestica* (F7AHC3).

Immunostaining

BHK cells were cultured in 8-well chambered cover glass (IWAKI, Tokyo, Japan). Intact cells were fixed with 4% PFA/PBS and washed with PBS twice. Permeablised cells were also fixed with 4% PFA/PBS, washed with PBS twice, and permealised by 0.1% Triton X-100/PBS. After fixation, we added 1:1000 rat anti-IZUMO antibody (KS64-125; gifted from Dr. Ikawa), rat anti-JUNO antibody (TH6; BioLegend) in PBS, and incubated for 3 hours. The secondary antibodies which were goat anti-rat IgG conjugated with Alexa Fluor 488 or Alexa Fluor 594 (Invitrogen) for IZUMO and JUNO and goat anti-rabbit IgG conjugated with Alexa Fluor 488 were diluted 1:500 in PBS. Immunofluorescence images were obtained using an inverted fluorescence microscope (IX-83; Olympus, Tokyo, Japan) equipped with spinning disk confocal

system (CSU-W1; Yokogawa Electric, Tokyo, Japan) and 488-nm and 561-nm LD lasers. Each experiment was repeated at least three times.

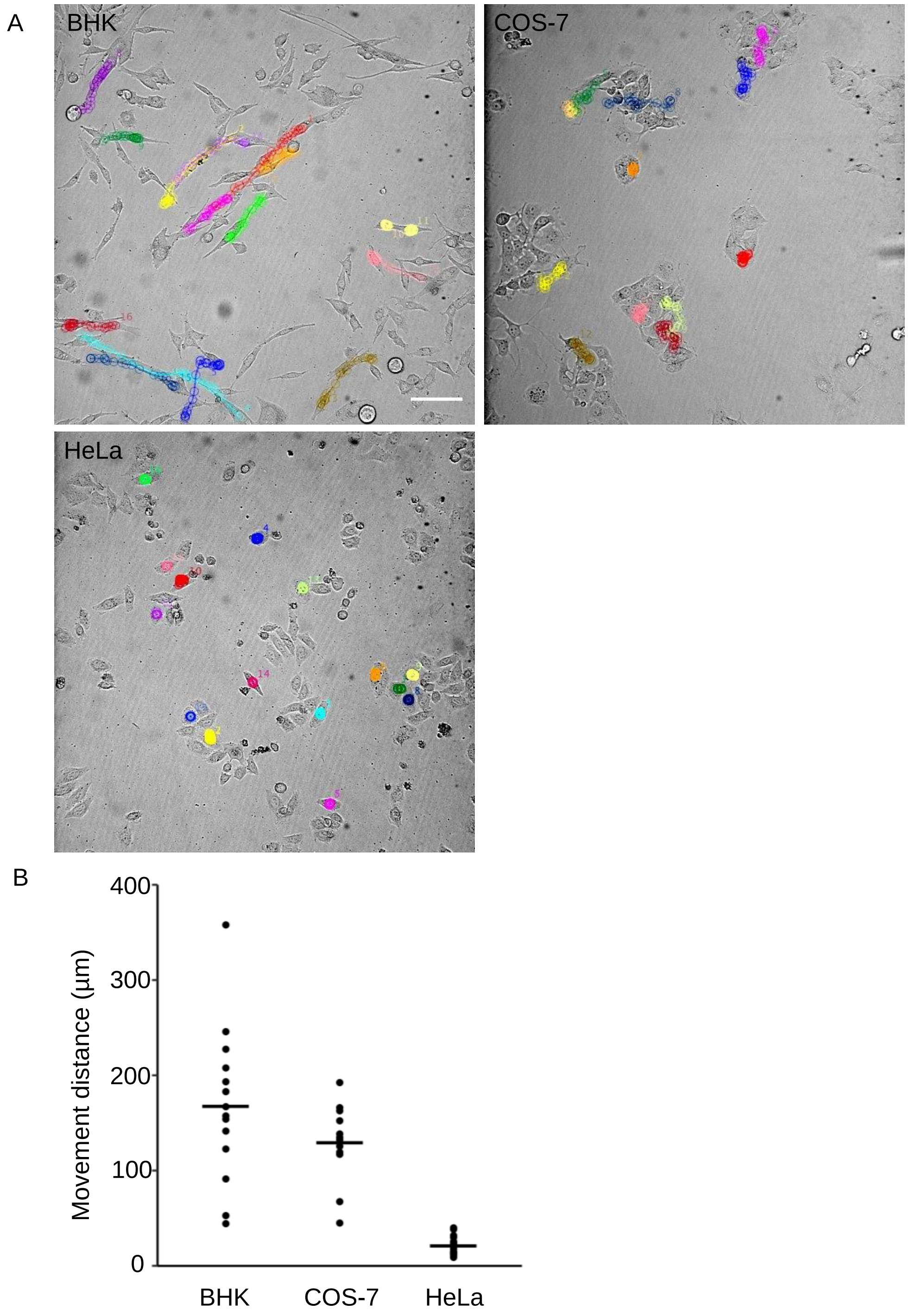

Figure S1 | Tracking of cultured cells

(A) Tracking of individual cultured baby hamster kidney (BHK), Cos-7, and HeLa cells using the Fiji software plugin MTrackJ. Bars: 50 µm. (B) Movement distance of cultured cells. Dots indicate the distance travelled by each cultured cell; bars indicate their averages.

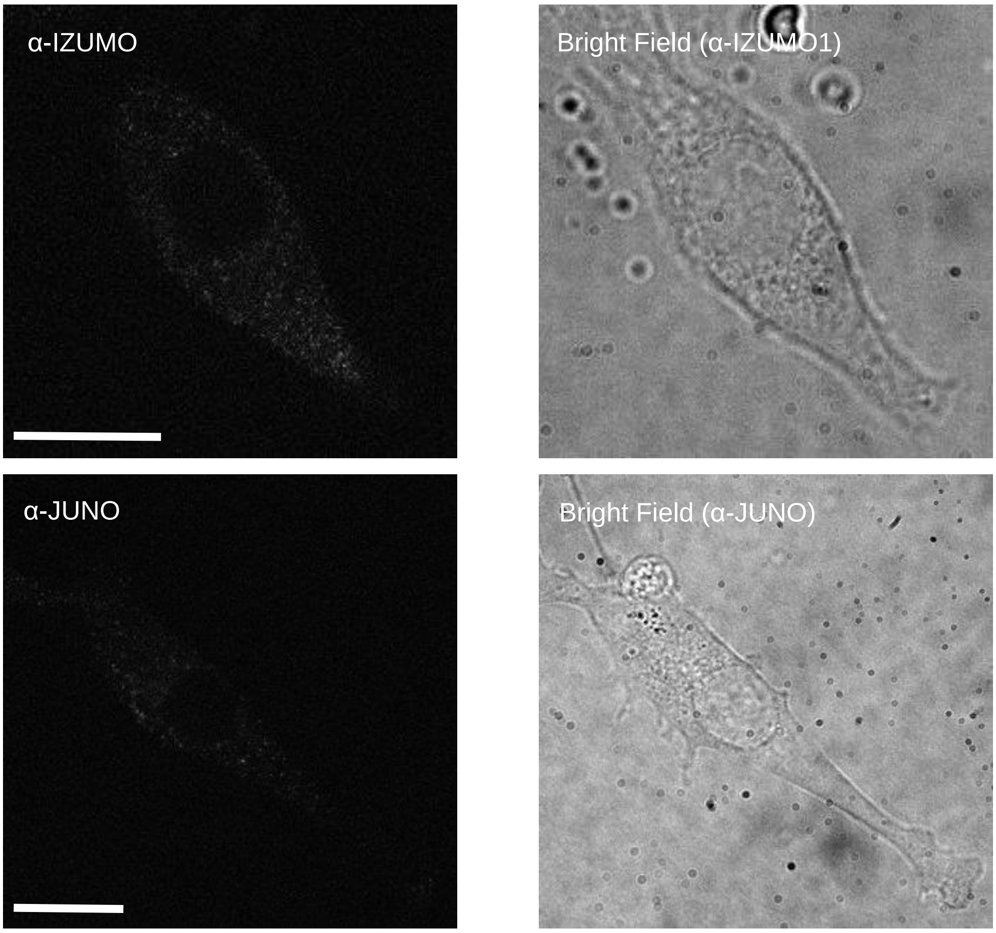

Figure S2 | Immunostaining of BHK cells using α-IZUMO1 and α-JUNO
Immunofluorescence of untransfected BHK cells as negative control. α-IZUMO1 (top) and α-JUNO (bottom) did not have affinity for BHK cells. Immunostaining and observation were performed as same as the case of transfected BHK cells (Figs. S3-S5). Bars: 10 µm.

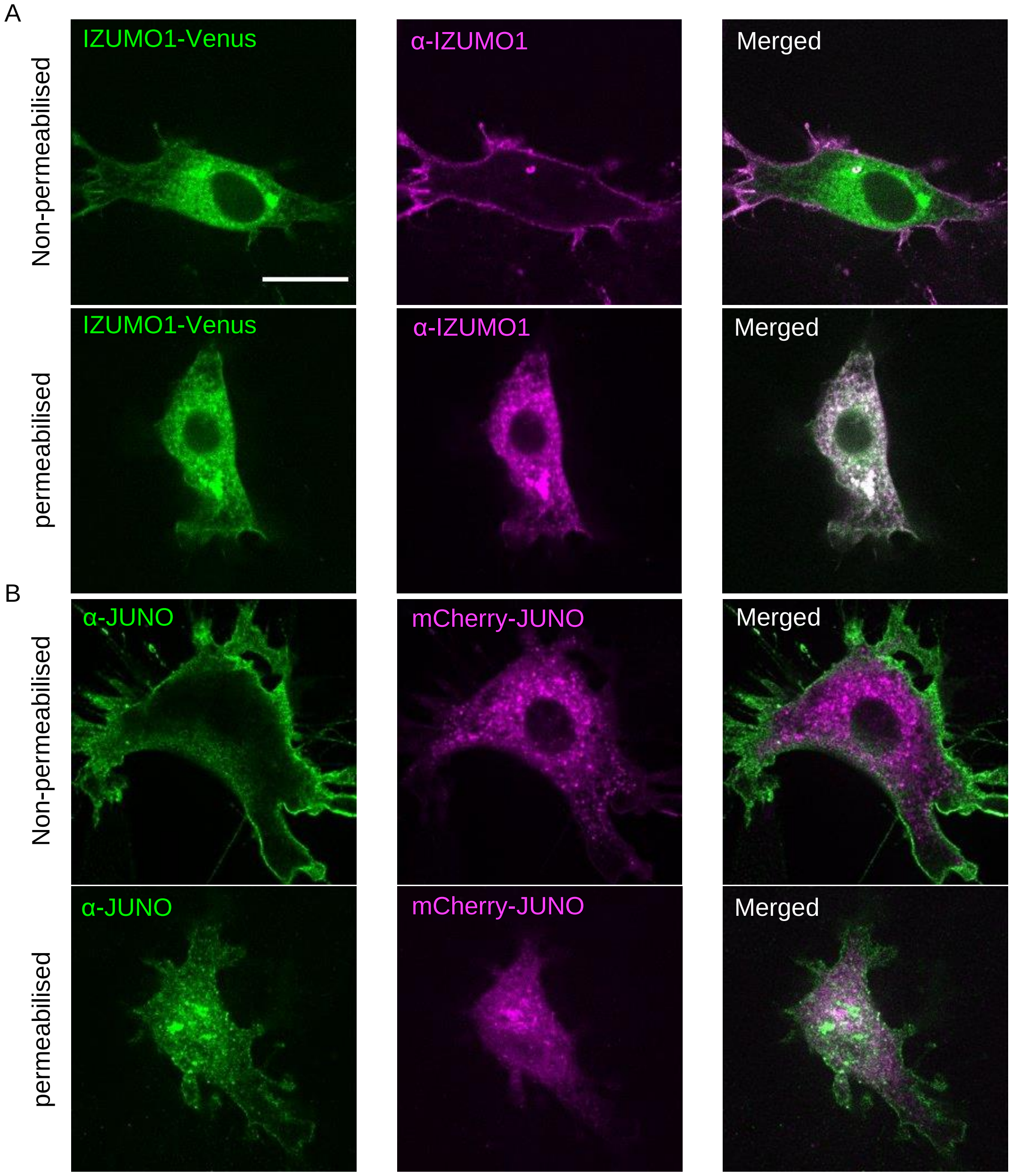

Figure S3 | Immunostaining of BHK cells expressing IZUMO1 and JUNO

(A) IZUMO1 was localised at the surface without permeabilisation (top). α-IZUMO1 and Venus were colocalised after permeabilization with tritonX-100 (bottom). α-IZUMO1 was labelled with α- rat IgG conjugated with Alexa Fluor 488. (B) JUNO was localised at the surface without permeabilisation (top). α-JUNO and mCherry were colocalised after permeabilization with tritonX-100. JUNO was also localised at the surface (bottom). α-JUNO was labelled with α- rat IgG conjugated with Alexa Fluor 594. Bar: 10 μm.

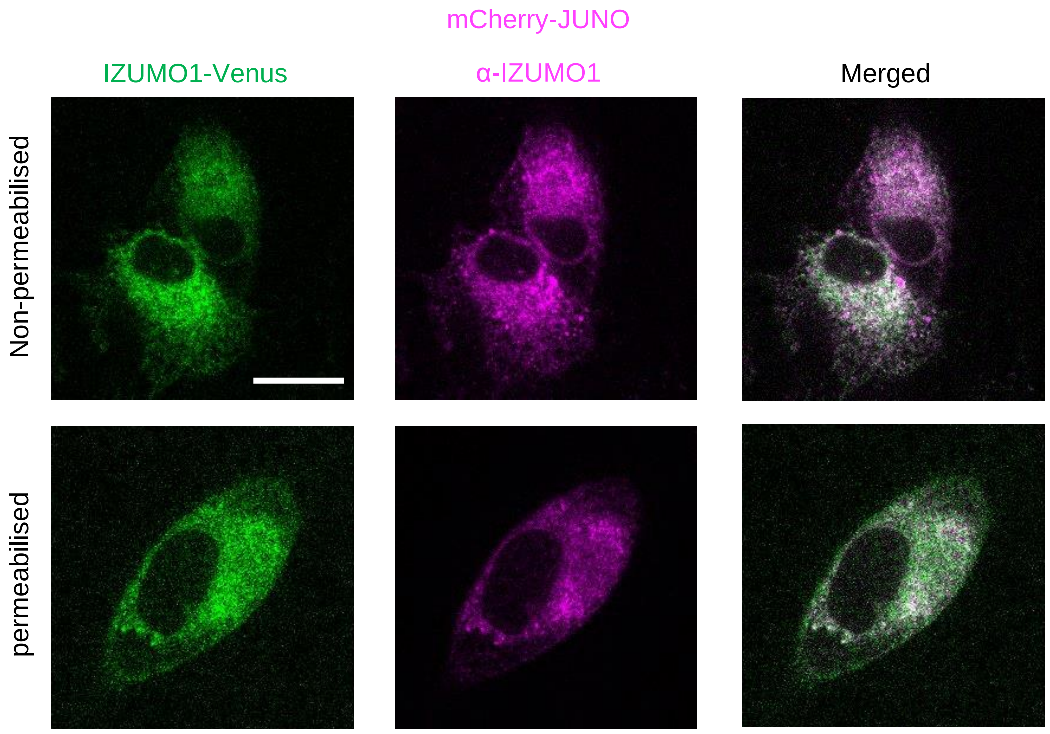

Figure S4 | Immunostaining of BHK cells co-expressing IZUMO and JUNO

Immunofluorescence of IZUMO-Venus and mCherry-JUNO co-expression at the surface. IZUMO was localised at ER without permeabilisation (top). α-IZUMO1 and Venus colocalised after permeabilization (bottom). Bar: 10 µm.

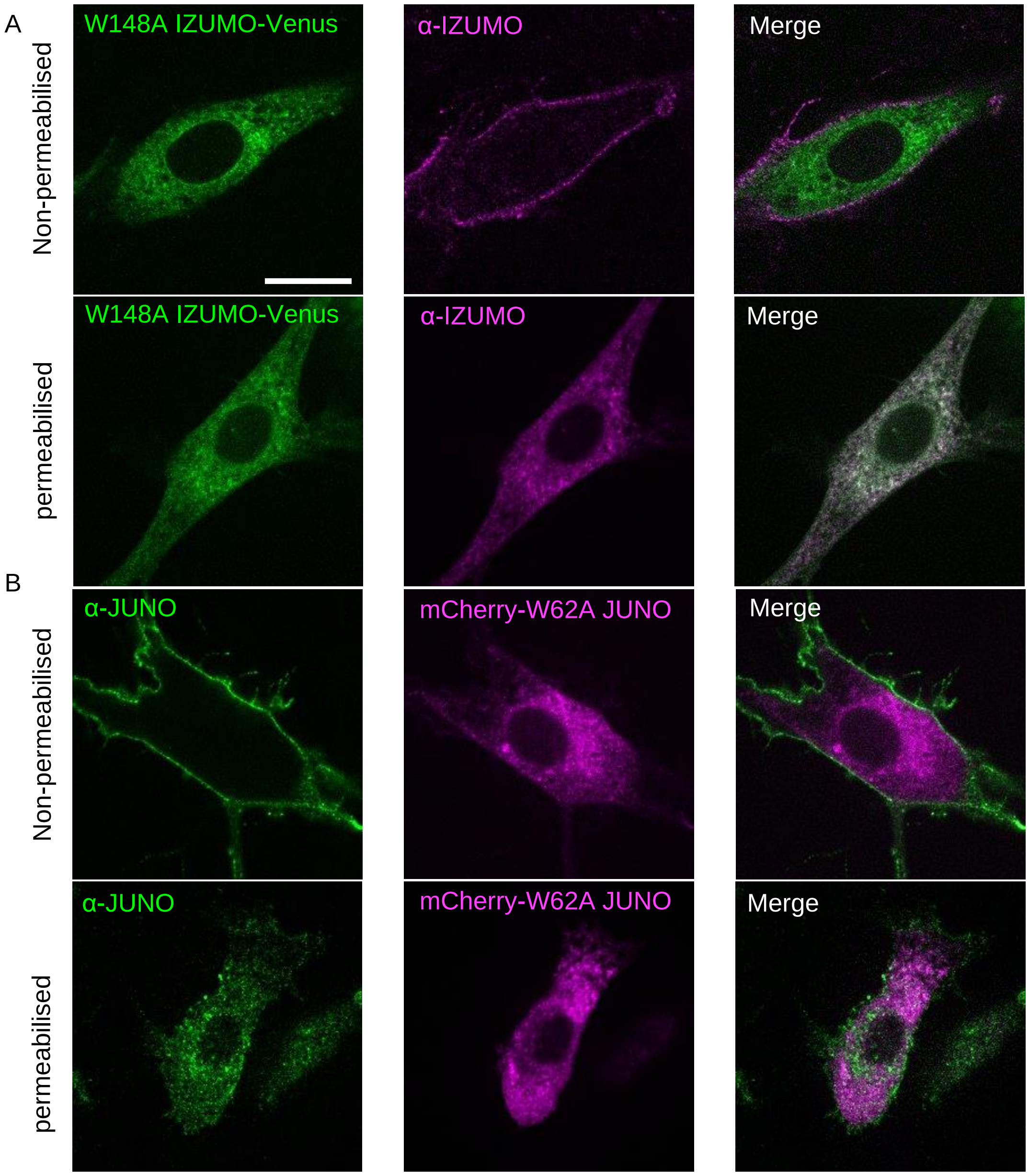

Figure S5 | Immunostaining of BHK cells expressing W148A IZUMO and W62A JUNO

(A) W148A IZUMO1 was localised at the surface without permeabilisation as well as IZUMO1-Venus (top). α-IZUMO1 and Venus were colocalised after permeabilization (bottom). α-IZUMO1 was labelled with α- rat IgG conjugated with Alexa Fluor 488. (B) JUNO was localised at the surface without permeabilisation (top). α-JUNO and mCherry were colocalised after permeabilization. JUNO was also localised at the surface (bottom). α-JUNO was labelled with α- rat IgG conjugated with Alexa Fluor 594. Bar: 10 µm.

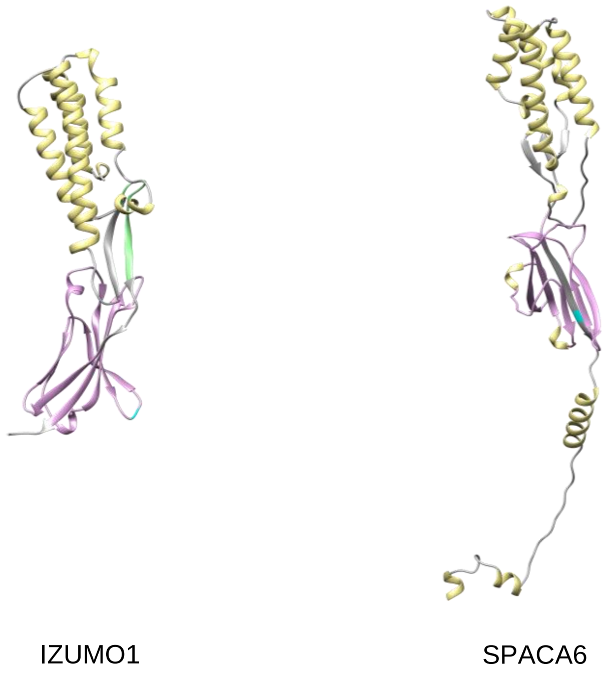

Figure S6 | IZUMO1 and SPACA6 structures
IZUMO1 was modelled using Chimera software based on amino acid sequence (Uniprot accession no. Q9D9J7). SPACA6 was modelled by AlphaFold. Both IZUMO1 and SAPCA6 encode a signal peptide, Ig-like domain (magenta), N-linked glycosylation sites (cyan), and type-I transmembrane domain[6,] SPACA6 is localized on the acrosomal cap of the sperm head[6,7] and has similar domain structures to IZUMO1 including four α-helix bundle.

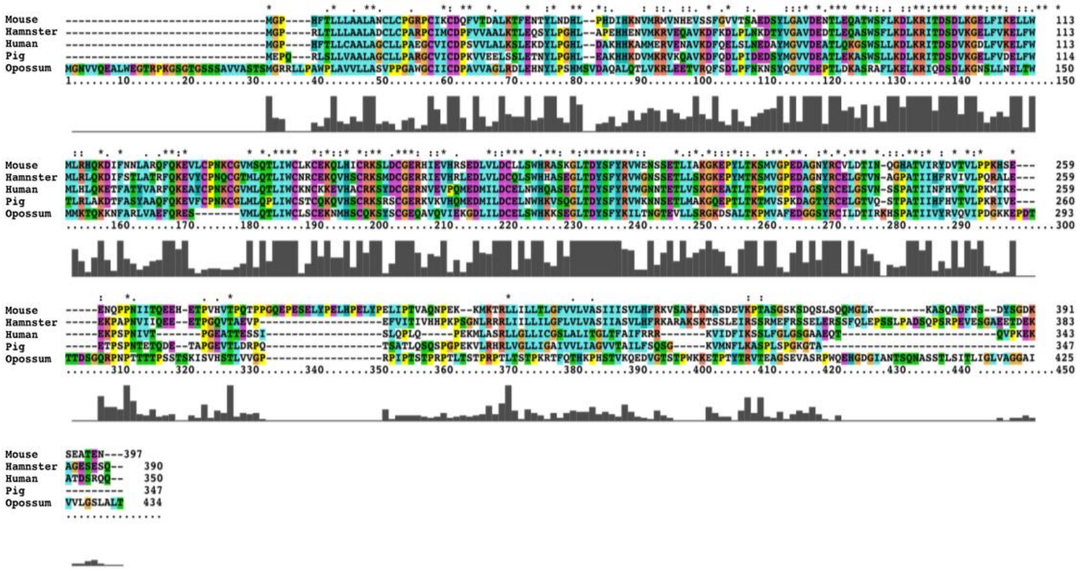

Figure S7 | Comparison of IZUMO1 amino acid sequence

Sequences were aligned using the Clustal X program. Parameters and colour codes were based on the Clustal X scheme. Amino acid sequences for entire IZUMO1 proteins were used as follows: mouse, *Mus musculus* (Uniprot accession no. Q9D9J7); human, *Homo sapiens* (Q8IYV9); golden hamster, *Mesocricetus auratus* (A0A1U7QS27); pig, *Sus scrofa* (A0A287BMD8), and opossum, *Monodelphis domestica* (H9H653).

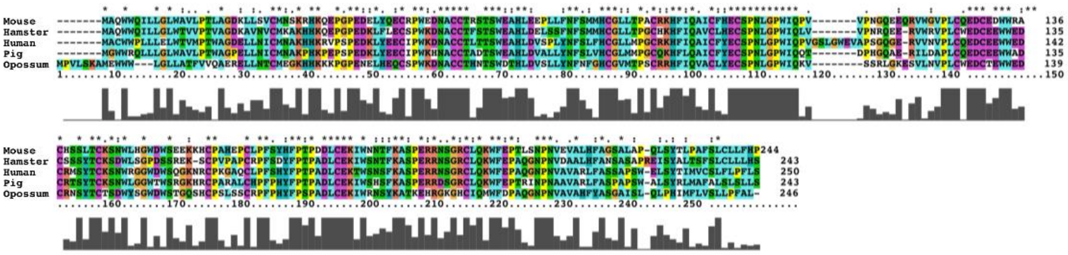

Figure S8 | Comparison of JUNO amino acid sequence

Sequences were aligned using the Clustal X program. Parameters and colour codes were based on the Clustal X scheme. Amino acid sequences for entire JUNO proteins were used as follows: mouse, *M. musculus* (Q9EQF4); human, *H. sapiens* (A6ND01); golden hamster, *M. auratus* (A0A1U7RC80); pig, *S. scrofa* (F1STK4), and opossum, *M. domestica* (F7AHC3).

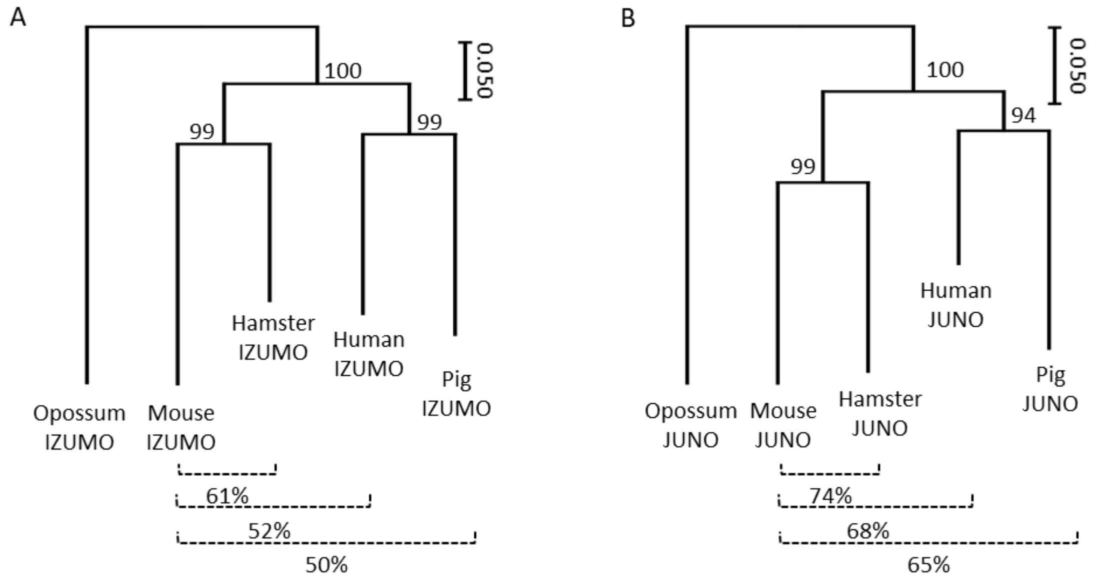

Figure S9 | Phylogenetic trees of IZUMO1 and JUNO

Neighbour-joining phylogenetic trees of IZUMO1 (A) and JUNO (B) were constructed using the MEGA X program. Bootstrap analysis was performed with 1,000 replicates. %: percent identity compared with mouse.

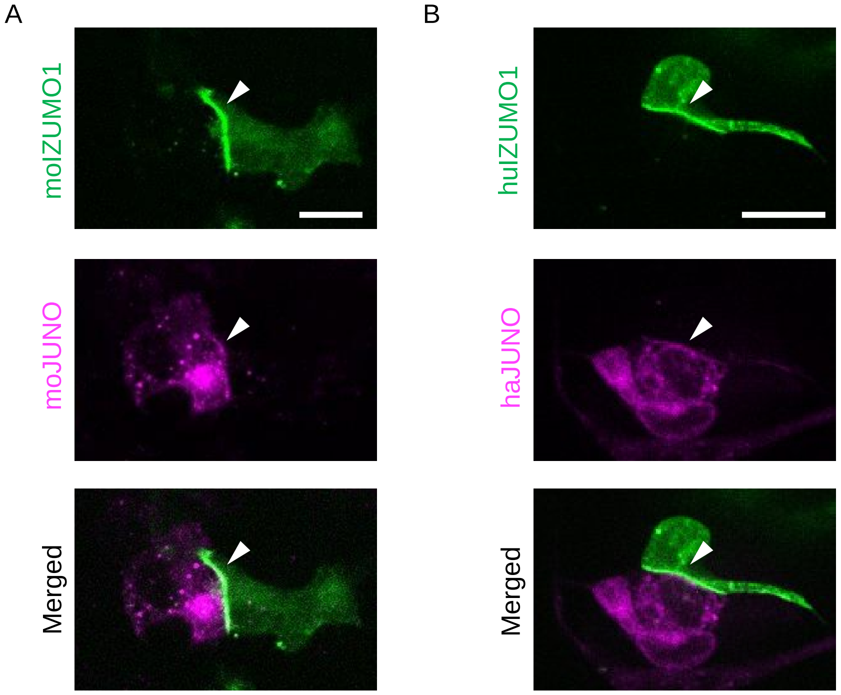

Figure S10 | Accumulation of human IZUMO1 and hamster JUNO in HEK293T cells

(A) Mixing of mouse IZUMO1-expressing cells (green) and mouse JUNO-expressing cells (magenta) induced accumulation. (B) Mixing of human IZUMO1-expressing cells (green) and hamster JUNO-expressing cells (magenta) also induced accumulation. Arrow heads indicate accumulation of IZUMO1 or JUNO. Bars: 20 μm.

Table S1 | Accumulation and translocation of W148 IZUMO1, W62A JUNO and SPACA6 in BHK cells

	Numbers of contacted cell-pairs	IZUMO1 accumulation	JUNO accumulation	IZUMO1 translocation	JUNO translocation
IZUMO1	31	0	0	0	0
vs	23	0	0	0	0
W62A JUNO	13	0	0	0	0
W148A	10	0	0	0	0
IZUMO1	15	0	0	0	0
vs JUNO	20	0	0	0	0
SPACA6	12	0	0	0	0
vs	4	0	0	0	0
JUNO	24	0	0	0	0

Table S2 | Accumulation and translocation of IZUMO1 and JUNO in conspecific and heterospecific cell combinations in BHK cells

	Numbers of contacted cell-pairs	IZUMO1 accumulation	JUNO accumulation	IZUMO1 translocation	JUNO translocation
moIZUMO1	23	18	8	10	4
vs	10	8	6	2	0
moJUNO	21	13	6	3	0
huIZUMO1	19	2	5	0	13
vs	18	6	4	0	12
huJUNO	14	6	6	0	8
haIZUMO1	16	10	4	11	12
vs	27	22	2	11	2
haJUNO	14	12	3	3	2
piIZUMO1	6	2	0	0	0
vs	11	4	0	0	0
piJUNO	24	4	0	0	0
moIZUMO1	13	9	0	0	0
vs	9	4	0	0	0
huJUNO	12	4	0	0	0
moIZUMO1	22	17	0	0	0
vs	5	4	0	0	0
haJUNO	13	10	0	0	0
moIZUMO1	4	0	0	0	0
vs	10	0	0	0	0
piJUNO	12	0	0	0	0
huIZUMO1	17	0	0	0	0
vs	17	0	1	0	0
moJUNO	16	0	0	0	0
huIZUMO1	9	0	0	0	0
vs	30	0	0	0	0
haJUNO	7	0	0	0	0

huIZUMO1	12	0	0	0	0
vs	19	0	0	0	0
piJUNO	8	0	0	0	0
haIZUMO1	5	2	1	0	0
vs	17	6	2	0	0
moJUNO	14	4	6	0	0
haIZUMO1	10	0	0	0	0
vs	39	2	0	0	0
huJUNO	9	0	0	0	0
haIZUMO1	8	0	0	0	0
vs	23	0	0	0	0
piJUNO	6	0	0	0	0
piIZUMO1	14	0	0	0	0
vs	24	0	0	0	0
moJUNO	26	0	0	0	0
piIZUMO1	37	18	17	0	14
vs	19	8	10	1	3
huJUNO	7	4	4	1	3
piIZUMO1	21	2	2	0	0
vs	24	3	2	0	0
haJUNO	39	6	2	0	0

Table S3 | Accumulation and translocation of IZUMO1 and JUNO in conspecific cell combinations in HEK293T cells

	Contact	IZUMO1 Accumulation	JUNO Accumulation	IZUMO1 translocation	JUNO translocation
moIZUMO1	41	39	22	10	3
vs	19	16	9	4	1
moJUNO	14	11	10	3	5
Total	74	66	41	17	9
		89%	55%	23%	12%

Table S4 | Accumulation and translocation of IZUMO1 and JUNO in heterospecific cell combinations in HEK293T cells

	Contact	IZUMO1 Accumulation	JUNO Accumulation	IZUMO1 translocation	JUNO translocation
huIZUMO1	52	6	1	0	0
vs	29	3	2	0	0
haJUNO	26	1	0	0	0
Total	107	10	3	0	0
		9.3%	2.8%	0%	0%

Table S5 | Sequences of oligonucleotide primers

Primer name	Sequence (5'-> 3')
GCS1_F	TCACAGGCCACCAAGCTTGGTACCATGGTGAACGCGATTTTAATGGCT
GCS1_R	GTGACCTCGAGCGGCCGCTTAACTCTCACGTAGTCTTTGTTTCCTCTG
GCS1Δ_F	AAGGCAAATACCGCGCATTG
GCS1Δ_R	AGGCATTCTTCGTTGCGGT
CD9_F	TCACAGGCCACCAAGCTTGGTACCATGCCGGTCAAAGGAGGTAG
CD9_R	GCTGCCCCCTCCACCTGAGACCATTTCTCGGCTCCTGC
mouseIZUMO11_F	TCACAGGCCACCAAGCTTGGTACCATGGGGCCGCATTTTACACTC
mouseIZUMO1_R	GCTGCCCCCTCCACCTGAGTTTTCTGTTGCCTCGCTCTTATCT
mouseJUNO_F	TCACAGGCCACCAAGCTTGGTACCATGGCACAGTGGTGGCAGA
mouseJUNO_R	GCTGCCCCCTCCACCTGAGGGATGGAACAACAGGCACAGA
mouseIZUMO1Δ_F	GATTGTGGAGAGCGCCACATAG
mouseIZUMO1Δ_R	AAGAACTTCCTTTTGGAACTGTCTAGC
humanIZUMO1_F	GATCTCGAGCTCAAGCTTGGTACCATGGGGCCGCATTTTACCC
humanIZUMO1_R	GCTGCCCCCTCCACCTGATTGTTGCCTCGAATCTGTGGC
humanJUNO_F	GATCTCGAGCTCAAGCTTGGTACCATGGCATGCTGGTGGCC
humanJUNO_R	TGATCTAGAGTCGCGGCCGCTTATCAGGAAAGGAACGGCAGGAAC
humanJUNO_F2	CCTCGCCCTTGCTCACCATAGCCCAGGTGGGCATG
humanJUNO_R2	GTGGATCTGGAGGTGGTGGAGGGGACGAGCTGCTCAACA
hamsterIZUMO1_F	GATCTCGAGCTCAAGCTTGGTACCATGGGGCCGCGTCTTAC
hamsterIZUMO1_R	GCTGCCCCCTCCACCTGATTGGCTTTCTGATTCCCCTGC
hamsterJUNO_F	GATCTCGAGCTCAAGCTTGGTACCATGGCACAGTGGTGGCAG
hamsterJUNO_R	TGATCTAGAGTCGCGGCCGCTTATCAGGAATGGAGCAGCAGGC
hamsterJUNO_F2	TCCTCGCCCTTGCTCACCATTCCTGCCACAGTGGGCAC

hamsterJUNO_R2	GTGGATCTGGAGGTGGTGGAGACAAGGCGGTCAACGTCTG
pigIZUMO1_F	GATCTCGAGCTCAAGCTTGGTACCATGGAGCCGCAGCGG
pigIZUMO1_R	GCTGCCCCCTCCACCTGAAGCAGTGCCTTTGCCAGG
pigJUNO_F	GATCTCGAGCTCAAGCTTGGTACCATGGGATGGTGGAGGCAGC
pigJUNO_R	TGATCTAGAGTCGCGGCCGCTTAGGACAACAAGCTCAGTGAAAGAGC
pigJUNO_F2	TCCTCGCCCTTGCTCACCATGGCCCAGGTAGGCAGCA
pigJUNO_R2	GTGGATCTGGAGGTGGTGGAGGTCCAGAACTCTTGAACATCTGTATG
Venus/mCherry/mTurquoise2_F	TCAGGTGGAGGGGGCAGCGGGGGGGGAGGTATGGTGAGCAAGGGCGAG
Venus/mCherry/mTurquoise2_R	GTGACCTCGAGCGGCCGCTTACTTGTACAGCTCGTCCATGCC
mCherry for JUNO_F	ATGGTGAGCAAGGGCGAGG
mCherry for JUNO_R	TCCACCACCTCCAGATCCACCACCTCCAGACTTGTACAGCTCGTCCATGCC
