# Supplementary material for: Live imaging-based assay for visualising species-specific interactions in gamete adhesion molecules
Source: Sci Rep. 2022 Jun 10;12:9609. doi: 10.1038/s41598-022-13547-w (PMC9187738; doi:10.1038/s41598-022-13547-w)
Supplement: Supplementary file 7 — Supplementary Legends. [file 41598_2022_13547_MOESM7_ESM.docx]

**Video 1.** GCS1-expressing cells showing bilateral fusion.

**Video 2.** E-cadherin accumulation at the contact interface of adjacent cells.

**Video 3.** IZUMO1 accumulation at the contact interface of JUNO-expressing cells.

**Video 4.** Simultaneous accumulation of IZUMO1 and JUNO at the contact interface of cells.

**Video 5.** IZUMO1 translocation to a JUNO-expressing cell.
